# Revisiting the Growth of Black Phosphorus in Sn-I Assisted Reactions

**DOI:** 10.3389/fchem.2019.00021

**Published:** 2019-01-28

**Authors:** Dongya Wang, Peng Yi, Lin Wang, Lu Zhang, Hai Li, Min Lu, Xiaoji Xie, Ling Huang, Wei Huang

**Affiliations:** ^1^Key Laboratory of Flexible Electronics (KLOFE), Institute of Advanced Materials, Nanjing Tech University, Nanjing, China; ^2^Shaanxi Institute of Flexible Electronics, Northwestern Polytechnical University, Xi'an, China

**Keywords:** black phosphorus, growth mechanism, tin iodide, chemical vapor transport, 2D materials

## Abstract

Black phosphorus, an emerging layered material, exhibits promising applications in diverse fields, ranging from electronics to optics. However, controlled synthesis of black phosphorus, particularly its few-layered counterparts, is still challenging, which should be due to the unclear growth mechanism of black phosphorus. Here, taking the most commonly used Sn-I assisted synthesis of black phosphorus as an example, we propose a growth mechanism of black phosphorus crystals by monitoring the reactions and analyzing the as-synthesized products. In the proposed mechanism, Sn_24_P_19.3_I_8_ is the active site for the growth of black phosphorus, and the black phosphorus crystals are formed with the assistance of SnI_2_, following a polymerization-like process. In addition, we suggest that all Sn-I assisted synthesis of black phosphorus should share the same reaction mechanism despite the differences among Sn-I containing additives. Our results shown here should shed light on the controlled synthesis of black phosphorus and facilitate further applications of black phosphorus.

## Introduction

With the rapid development of two-dimensional materials, orthorhombic black phosphorus (BP), assembled by puckered phosphorus layers of interlinked six-membered rings via van der Waals interactions, recently has attracted much research enthusiasm due to its layer-number-dependent properties (Hirsch and Hauke, 2018; Liu H. et al., 2018; Liu Y. et al., 2018). Specifically, few-layered BP possesses tunable band gap, ranging from 0.3 to 2.0 eV (Xia et al., 2014), and high carrier mobility (~1,000 cm^2^V^−1^s^−1^) (Li et al., 2014), which makes it promising for diverse applications, including field effect transistor, battery, sensor, and electrocatalyst (Liu et al., 2015; Zhang Y. et al., 2017; Hu et al., 2018). Despite the outstanding properties of BP and the recent inspiring studies, the exploration of the properties of BP and corresponding applications are still constrained to some extent because of the difficulties in synthesizing BP and its layered counterparts (Hu et al., 2018; Zhang et al., 2018).

In fact, BP is a thermodynamically stable form of phosphorus but does not exist naturally. Formerly, BP was synthesized by transforming red phosphorus or white phosphorus under harsh conditions (Bridgman, 1914, 1935, 1948). It was until 2007 that a simple synthetic approach based on Sn-I assisted chemical vapor transport reactions was developed for producing BP crystals in high quality and high yield (Lange et al., 2007). In this method, BP is synthesized, in an evacuated ampoule, by programmed heating of red phosphorus with the mineralizers (e.g., Sn/SnI_4_). It should be emphasized that the mineralizers, particularly Sn and I elements, are decisive in this chemical vapor transport based method. Currently, this Sn-I assisted method is the most commonly used strategy for the large-scale preparation of high quality BP crystals, which indeed boosts both the scientific research and technological development of BP (Nilges et al., 2008; Köpf et al., 2014). Nevertheless, controlled synthesis of BP, particularly the synthesis of uniform thin BP film (few-layered BP), has not been achieved by chemical vapor transport based methods yet (Yang et al., 2015; Smith et al., 2016).

In order to realize controlled synthesis of BP, it is crucial to fully understand the corresponding formation mechanisms. Unfortunately, the formation mechanisms of BP in the Sn-I assisted method are still unclear, although several efforts have been devoted to revealing the detailed formation processes (Zhao et al., 2016a,b; Li et al., 2017; Zhang Z. et al., 2017; Shriber et al., 2018). Furthermore, the currently proposed formation mechanisms are different from each other, although they share some opinions. For example, in 2016, a molten alloy based mechanism was proposed (Zhao et al., 2016a). In this mechanism, BP crystals were believed to precipitate from the molten alloy of red phosphorus and metallic Sn when the temperature decreased. At almost the same time, a phase-transfer mechanism was proposed, in which BP crystals were proposed to be transformed from Hittorf's phosphorus with the assistance of a certain P-Sn-I ternary compound (Zhang Z. et al., 2017). Later on, BP crystals were suggested to grow, obeying a vapor-solid-solid mechanism, through the diffusion of excess P atoms from a Sn_24_P_22−x_I_8_ (x ≈ 2.7) intermediate compound (Li et al., 2017). In addition to the studies based on experimental observations, another BP formation pathway was advised recently according to first-principle calculations (Shriber et al., 2018). According to the density functional theory calculations, BP is favorably formed, in the presence of Sn-I containing mineralizer at high temperature and pressure, by a series of additions of P_4_ molecules in a polymerization-like process. Collectively, more efforts are desired to elucidate the formation mechanisms of BP crystals in the Sn-I assisted synthetic method.

In this study, taking the previous studies into account, we propose a new formation mechanism of BP crystals in the Sn-I assisted reaction according to a series of reaction observations and product characterization. In our proposed mechanism, the Sn and I containing mineralizers first decompose, forming SnI_2_ and Sn, and then the decomposed compounds react with phosphorus vapor at elevated temperature to form Sn_24_P_19.3_I_8_. Subsequently, the Sn_24_P_19.3_I_8_ molecules transport to and partially deposit at the zone with slightly low temperature (~550–500°C) during the cooling stage, generating the growth sites for BP crystals. The Sn_24_P_19.3_I_8_ molecules at the deposition zone can decompose to release SnI_2_, P_4_, and even P_2_ at the temperature of ~550–500°C, and thus are highly active. Meanwhile, the remaining SnI_2_ in the gas phase, functioning as a mineralizer, can transport P_4_ molecules to the growth sites by forming a Sn-P-I intermediate compound, yielding BP crystals in a polymerization-like process. In addition, we suggest that the Sn-I assisted synthesis of BP crystals should have the same growth processes, although different Sn and I containing additives can be used as mineralizers. We believe that this study can improve the understanding of BP synthesis, and facilitate controlled synthesis of BP for future applications.

## Experimental Section

### Materials

Tin powder (Sn, 99.99%) and iodine granule (I_2_, AR, 99.8%) were purchased from Aladdin (Shanghai, China). Red phosphorus (99.999%, metals basis) was purchased from Alfa Aesar. Acetic acid (AR), acetic anhydride (AR), toluene (AR), and other chemicals were purchased from Shanghai Lingfeng Chemical Reagent Co., Ltd. All chemicals were used as received without further purification unless otherwise noted.

### Synthesis of SnI_4_, SnI_2_, Sn_4_P_3_, Sn_24_P_19.3_I_8_, SnIP, and Black Phosphorus (BP)

#### Synthesis of SnI_4_ (Köpf et al., 2014)

To a mixture of acetic acid (25 mL) and acetic anhydride (25 mL), 0.5 g Sn powder and 2 g I_2_ were added. The resulting mixture was refluxed at 120°C for 90 min, during which the Sn powder completely disappeared. After the mixture was cooled to room temperature, orange-colored SnI_4_ crystals appeared and were collected. The obtained SnI_4_ crystals were recrystallized in chloroform for further use.

#### Synthesis of SnI_2_

Typically, a mixture of SnI_4_ (1.2956 g) and Sn (246.2 mg) powders (molar ratio: 1:1) was loaded in an evacuated silica ampoule (~10 cm in length and ~10 mm in inner diameter). Subsequently, the ampoule was heated to 400°C within 1 h in a muffle furnace, annealed for 5 h, and cooled down to room temperature naturally. The SnI_2_ powders, on the wall of the silica ampoule, were collected for further use.

#### Synthesis of Sn_4_P_3_

A mixture of grounded red phosphorus powder (72 mg) and tin powder (364 mg) was sealed in an evacuated silica ampoule (~10 cm in length and ~10 mm in inner diameter). The sealed ampoule was then annealed at 400°C for 8 h in a muffle furnace, and cooled to 200°C in 2 h. Finally, the ampoule was naturally cooled down to room temperature and the resulting Sn_4_P_3_ was collected.

#### Synthesis of Sn_24_P_19.3_I_8_ and SnIP

A mixture of tin powder, red phosphorus powder, and I_2_, with stoichiometric ratio, was sealed in an evacuated silica ampoule. Subsequently, the sealed ampoule was heated to 400°C within 40 min, kept for 10 h, and slowly cooled down to room temperature in 75 h. The ternary compounds were then collected from the silica ampoule.

#### Synthesis of Black Phosphorus (BP)

In a typical experiment, a mixture of red phosphorus (300 mg) and mineralizers (SnI_4_: 6 mg, Sn: 12 mg) was sealed in an evacuated silica ampoule (~10 cm in length and ~10 mm in inner diameter). The ampoule was placed horizontally in the center zone of a muffle box furnace with a viewing window. It should be noted that the temperature at the center of the furnace is slightly different from that near the wall. The temperature of the furnace was increased from room temperature to 650°C in 1 h, and then decreased to 550°C within 1 h. Afterwards, the temperature was further decreased to 500°C in 8 h, and then to 200°C in 4 h. Finally, the furnace was turned off for natural cooling to room temperature. After the reaction, BP crystals were collected from the ampoule, washed with hot toluene (~60°C) for three times until the toluene was colorless, dried at N_2_ atmosphere and stored at ambient environment.

BP crystals were also prepared in the presence of different mineralizers, such as Sn_24_P_19.3_I_8_ (21.6 mg), SnIP (10.6 mg), a mixture of Sn and I_2_ (Sn: 4.5–14 mg, I_2_: 4.8 mg) and a mixture of SnI_2_ and Sn (SnI_2_: 7.1 mg, Sn: 11.2 mg), by keeping other conditions identical. Notably, despite the differences among varied mineralizers, the molar amount of I element was kept the same (0.038 mmol) when different mineralizers were used. All synthesis reactions, including the control experiments, were carried out under the same temperature program unless otherwise noted. To record the reaction processes, photographs were taken from the viewing window of the furnace at different stages during the reaction.

## Characterization

Powder X-ray diffraction (XRD) patterns were recorded using a Rigaku Smartlab (9 kW) X-ray diffractometer with Cu Kα radiation (λ = 1.5406 Å). Raman spectra were acquired on a HR Evaluation spectrometer (Horiba, Confocal Raman Microscope) with a 532 nm laser. Thermogravimetric (TG) analysis was performed on a METTLER TGA2 under the N_2_ atmosphere at a heating rate of 10°C/min. Scanning electron microscopy (SEM) image and corresponding energy dispersive X-Ray spectroscopy (EDX) analysis were carried out in a JSM-7800F (JEOL) scanning electron microscope. Low resolution transmission electron microscopy (TEM) measurements were carried out on a JEM-1400 transmission electron microscope at an acceleration voltage of 120 kV. High resolution TEM, dark-field scanning transmission electron microscopy (STEM), and corresponding EDX measurements were conducted on a 2100F transmission electron microscope at an acceleration voltage of 200 kV. Photographs were taken by a digital camera.

## Results and Discussion

### Characterization of BP Crystals

**Figure 1:**
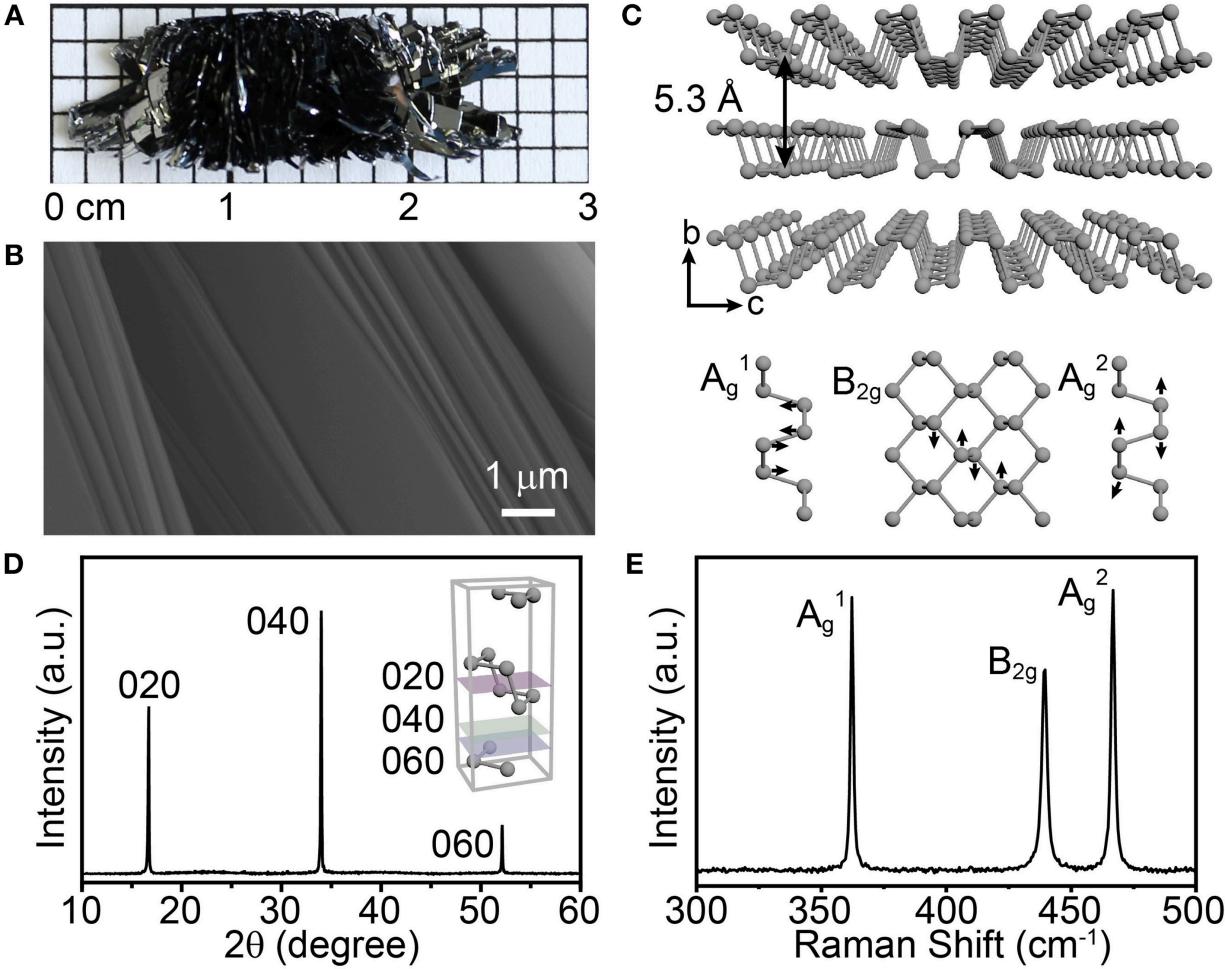
**(A)** Photo of the as-prepared bulk BP crystals. **(B)** SEM image showing the layered structure of the BP crystal. **(C)** Schematic illustrations of the atomic structure of orthorhombic BP and corresponding vibrational modes in Raman spectroscopy. **(D)** Powder XRD pattern of the obtained BP crystals. Inset: schematic illustration of a crystal cell of orthorhombic BP and its crystal planes. **(E)** Raman spectrum of the as-prepared BP.

Prior to studying the growth process of BP crystals, we first confirmed that high quality BP crystals can be obtained under our synthetic conditions. On a separate note, considering that the mixture of SnI_4_ and Sn is one of the most commonly used mineralizers, we here chose the SnI_4_/Sn mixture as a representative mineralizer in the following sets of experiments. As shown in Figure 1A, the obtained bulk BP crystals present a flower-like (radial) shape, with many sheetlike branches, on a centimeter scale and black color with metallic luster. Meanwhile, as revealed by the scanning electron microscopy (SEM) image, the BP crystal has a layered structure (Figure 1B), exhibiting the essential feature as a layered material (Figure 1C, upper panel). Further powder X-ray diffraction (XRD) analysis shows that the as-synthesized BP crystals give three main diffraction peaks at ~16, 34, and 52° (Figure 1D). The XRD pattern reveals that the BP crystals are in orthorhombic phase with excellent crystallinity, while the three diffraction peaks indicate that the crystals grow in a highly oriented manner. Consistently, in the Raman spectrum, three characteristic peaks, at ~361, 438, and 466 cm^−1^, were observed (Figure 1E), which can be attributed to the characteristic A_g_^1^, B_2g_, and A_g_^2^ modes in the orthorhombic BP lattice (Figure 1C, lower panel) (Guo et al., 2015; Ribeiro et al., 2018; Wang et al., 2018). In addition, high resolution transmission electron microscopy analysis of the exfoliated BP sheets, together with the energy dispersive X-Ray spectroscopy (EDX) analysis, shows the good crystallinity and purity of the as-synthesized BP crystals (Figure S1). Collectively, orthorhombic BP crystals, with excellent quality, can be successfully synthesized in the presence of the SnI_4_/Sn mineralizer under our reaction conditions.

### Influence of Mineralizer Composition on the Synthesis of BP Crystals

**Figure 2:**
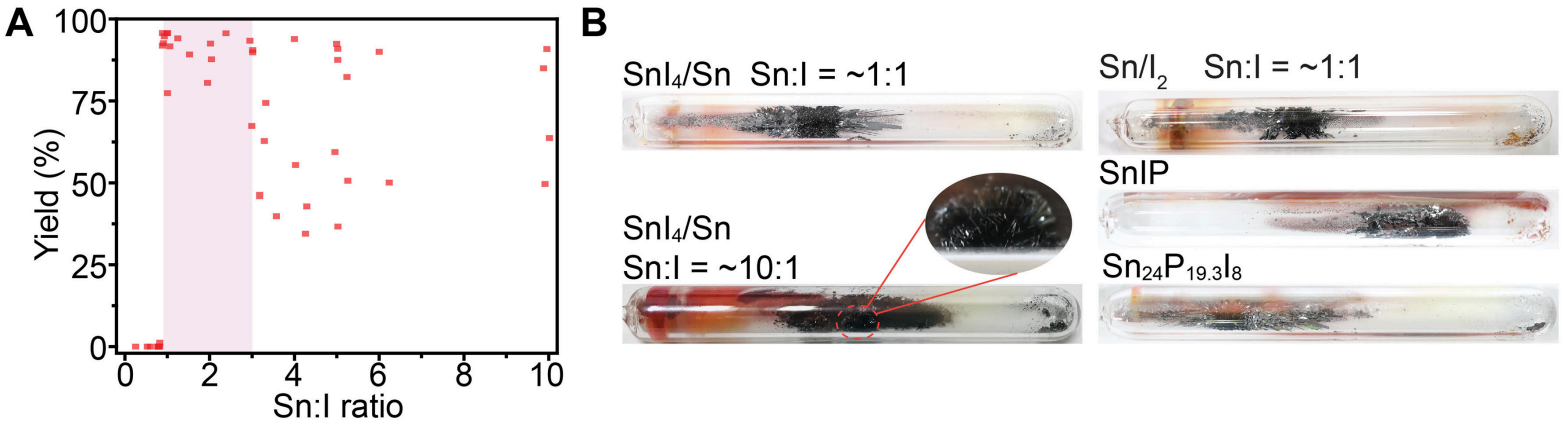
**(A)** Distribution of the synthetic yield of BP crystals against Sn:I ratio in the SnI_4_/Sn assisted reaction. **(B)** Photos of BP crystals in ampoules obtained in the presence of different mineralizers.

In order to understand the functions of the Sn-I containing mineralizers in the reactions, we first systematically tuned the molar ratio of Sn and I elements in the mineralizer by changing the amount of Sn. We found that the molar ratio of Sn:I had a threshold at ~0.9:1, below which almost no BP crystals can be synthesized (Figure 2A and Figure S2). It should be mentioned that a similar threshold of the Sn:I molar ratio (2:1) was also observed by a previous report (Li et al., 2017), although the reported ratio is slightly different from that we found here. The difference may be due to variations in reaction conditions, including heating process, silica tube, and furnace. In particular, BP crystals with high quality can be obtained in high yield (~90%) when the molar ratio of Sn:I is in the range from ~0.9:1 to 3:1 (Figures 2A,B). Further increase of the Sn:I ratio can also produce BP crystals, but the yield of BP crystals declines in most cases. For example, when the Sn:I ratio was set at ~10:1, small-sized BP crystals, scattered on the inner wall of the ampoule, were observed with a large number of side products (Figure 2B). These results indicate that the optimal molar element ratio of Sn and I, for preparing high quality BP crystals in high yield under our conditions, is in the range from ~0.9:1 to 3:1, when the SnI_4_/Sn mineralizer is used.

To check whether the Sn:I ratio for BP synthesis is dependent on the types of the Sn-I containing mineralizers, we then employed other commonly used Sn-I containing mineralizers, such as Sn/I_2_ and SnI_2_/Sn (Figure S3), to synthesize BP crystals under the same reaction conditions. As expected, high quality BP crystals can be successfully prepared in the presence of Sn/I_2_ or SnI_2_/Sn mineralizer if the Sn:I ratio was kept in the range from ~0.9:1 to 3:1 (Figure 2B and Figure S4). If the Sn:I ratio was set smaller than ~0.9:1, the reproducibility of the reaction became poor and few BP crystals were obtained in most cases (Figure S5). To further verify the Sn:I ratio, two ternary Sn-P-I compounds, SnIP (Figure S6) and Sn_24_P_19.3_I_8_ (Figure S7), were applied as mineralizers (Shatruk et al., 1999; Pfister et al., 2016). It should be mentioned that the Sn:I ratio of each Sn-P-I compound is near either the upper or lower limit of the determined range. Similar to other mineralizers, both SnIP and Sn_24_P_19.3_I_8_ can yield centimeter-sized high quality BP crystals (Figure 2B) under the same reaction conditions, validating the determined optimum range of Sn:I ratio.

Collectively, these results reveal that the mineralizers, despite the different chemical compositions such as SnI_4_/Sn, Sn/I_2_, SnI_2_/Sn, SnIP, and Sn_24_P_19.3_I_8_, can facilitate the growth of BP crystals if a proper molar ratio of Sn and I elements is applied. Furthermore, according to these results, we can suppose that all Sn-I containing mineralizers should facilitate the formation of BP crystals through the same way.

### Growth of BP Crystals and Transformation of Mineralizer

**Figure 3:**
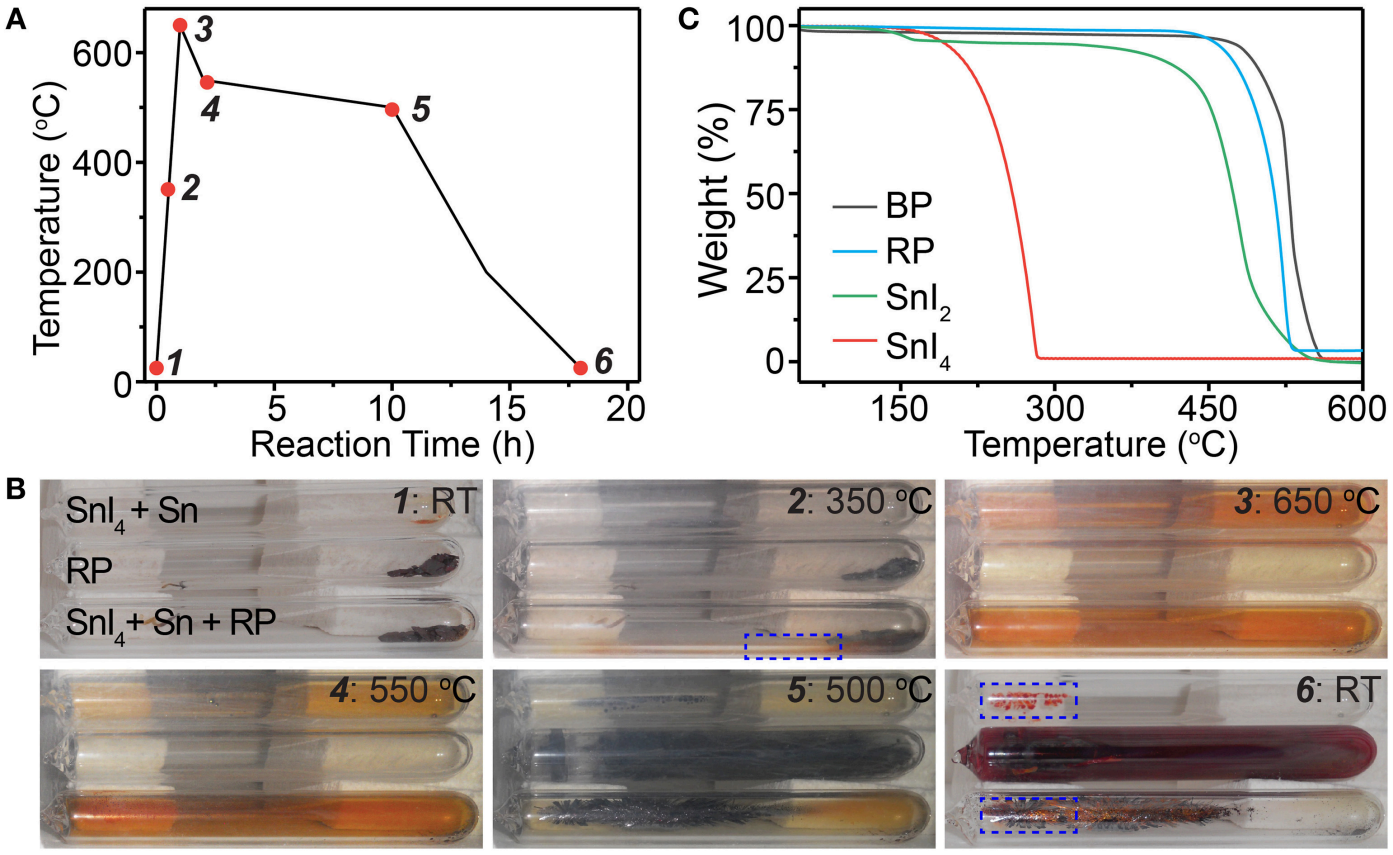
**(A)** Typical time dependent temperature profile for synthesizing BP crystals and **(B)** corresponding *in situ* photos taken during the synthesis. The red dots in the profile indicate the stages when the photos are taken. **(C)** Thermogravimetric profiles of different compounds. Note that RT and RP denote room temperature and red phosphorus, respectively.

Next, we monitored the synthesis of BP crystals (Figure 3 and Figure S8), by taking *in situ* photos at different stages during the reaction as marked in Figure 3A, to reveal the functions of the mineralizer. For comparison, pure red phosphorus and the SnI_4_/Sn mineralizer were also sealed solely in ampoules and monitored under the same conditions. As shown in Figure 3B1, the solid reactants initially were placed at the right side of the sealed ampoules. With the increase of the temperature, the SnI_4_/Sn mineralizer began to react with red phosphorus, exhibiting some mineralization effects at ~350°C (Figure 3B2, dotted box). When the temperature reached 650°C, almost all reactants including the mineralizer became vapor, leaving small amount of reactants at the original zone, and the whole ampoule exhibited orange color (Figure 3B3). Notably, the same orange color was also observed in the ampoule containing only the SnI_4_/Sn mineralizer, indicating the orange color comes from the vapor of mineralizer. Finally, the BP crystals appeared at the other side of the ampoule during the decrease of temperature, while some solid remained at the zone where the initial reactants were placed (Figures 3B5,B6). Interestingly, in the ampoules containing only the SnI_4_/Sn mineralizer, some orange-red colored solid appeared at almost the same position where BP crystals grew (Figure 3B6, dotted boxes). In stark contrast, in the absence of the mineralizer, the red phosphorus finally deposited randomly in the ampoules. Furthermore, we observed similar growth processes, with slight differences, when different types of mineralizers, such as Sn/I_2_, SnIP, and Sn_24_P_19.3_I_8_, were used (Figures S9–S11). These results show that the Sn-I containing mineralizer may first undergo transformation to form certain compounds and then react with phosphorus vapor to help the formation of BP crystals during the reaction.

To gain more insights into the growth process of BP crystals, we then tried to identify the transformation of mineralizer during the reaction. According to the thermogravimetric (TG) analysis (Figure 3C) and *in situ* photos (Figure 3B), all reactants, except Sn, should become vapor or decompose at 650°C. Comparing the status of pure I_2_, SnI_4_, and SnI_2_ at 650°C (Figure S12) with the observation shown in Figure 3B, we can deduce that the mineralizer should decompose to form gaseous SnI_2_ that exhibits orange color during the reaction. This deduction is also consistent with the previous report in which SnI_2_ is believed to be the most thermodynamically stable species in the reaction system at 650°C (Li et al., 2017). Regarding the material remaining at the zone where the initial reactants are placed at 650°C, we believe that it is liquid Sn because the boiling point of Sn is ~2,600°C (Dean, 1999). In addition, other compounds, including the common Sn-P and Sn-P-I compounds, cannot remain intact at such high temperature (~650°C) according to the TG analysis (Figure 3C and Figure S13). Accordingly, in the Sn-I assisted synthesis of BP crystals, we suggest that the Sn-I containing mineralizer should form SnI_2_ and Sn during the reaction.

To further verify the transformation of the mineralizer, we heated the SnIP and Sn_24_P_19.3_I_8_ compounds under the same reaction conditions and then analyzed the final compounds (Figure S14). As expected, SnI_2_ was observed at the place away from the zone where initial compounds were placed. Meanwhile, Sn_4_P_3_, formed by the reaction of Sn and P during the cooling process, appeared at the zone which originally contains the Sn-I-P compounds. In addition, we also found the presence of SnI_2_ on the as-prepared BP crystals. In this set of experiments, the BP crystals, prepared with the assistance of various mineralizers, were collected from the ampoule and then immersed into hot toluene. The toluene, with light yellow color (Figure S15), was then distilled, producing a few powders. As characterized by Raman spectroscopy, the powders can be identified as SnI_2_ (Figure S15).

Taken together, in the Sn-I assisted synthesis of BP crystals, the Sn-I containing mineralizer should first form sufficient SnI_2_. Subsequently, the gaseous SnI_2_ can react with gaseous phosphorus, forming a certain active Sn-P-I intermediate, and assist the formation of BP crystals.

### Active Site Identification and Characterization

**Figure 4:**
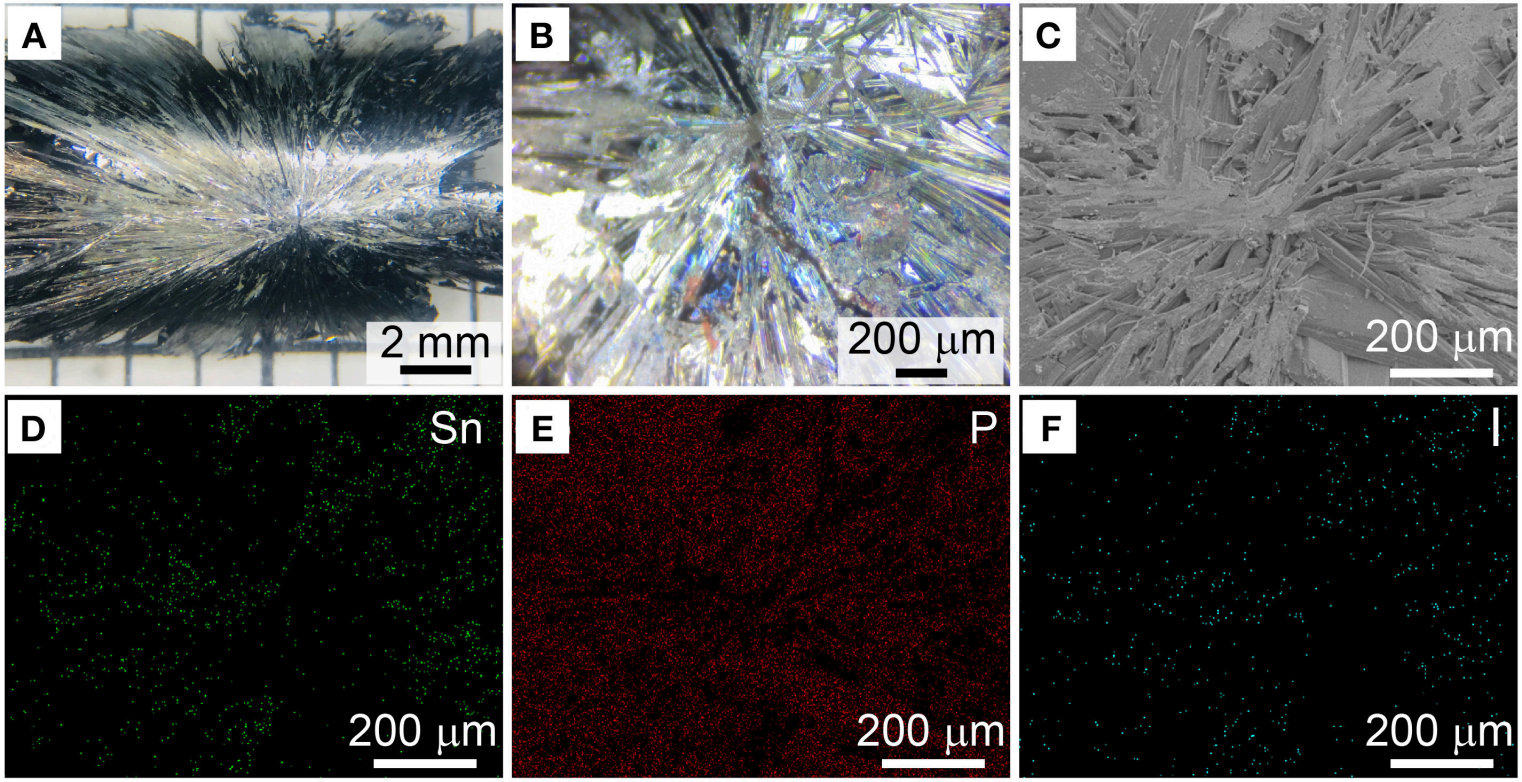
**(A)** Representative photo of the as-obtained BP crystals with an intersection point. **(B)** Corresponding enlarged photo of the intersection point. **(C)** SEM image of a randomly selected intersection point. **(D–F)** Corresponding elemental mapping of the intersection point shown in **(C)**.

In the following set of experiments, we intended to figure out the gaseous Sn-P-I intermediate and growth details of BP crystals by both monitoring the reaction and characterizing the as-synthesized BP crystals. Actually, we observed that BP crystals grew from the bottom of the ampoule at the cooling stage (mainly from 550 to 500°C) in reactions (Figure 3 and Figures S8–S11), and the growth of the crystals apparently exhibited an epitaxial manner. After the reaction, we collected the BP crystals from the ampoule tube and checked their back side which formerly attached on the wall of the ampoule. As shown in Figures 4A,B, a distinct intersection point with clear trails can be typically observed, indicating that the point is the starting point for growing BP crystals. Thus, we here suggest that the intersection point should be the active site for the growth of BP crystals. It should be mentioned that similar intersection points were also observed and considered as the nucleation sites for BP growth in previous studies (Zhao et al., 2016b).

Consequently, we analyzed the composition of the intersection point by energy dispersive X-Ray spectroscopy (EDX). At the intersection point of the as-prepared BP crystal, the molar ratio of Sn:I was determined as ~0.6:1 by EDX (Figure S16). After thoroughly washed by hot toluene, as exampled in Figures 4C–F, the Sn:I ratio at the intersection point was found as ~3.1:1 (Figure S17). Similarly, for the BP crystals obtained in the presence of Sn_24_P_19.3_I_8_ as a mineralizer, the Sn:I ratio at the intersection point was found as ~2.9:1 after toluene washing (Figure S18). Taking previous studies into account (Zhang et al., 2016; Li et al., 2017), we believe that the Sn_24_P_19.3_I_8_ compound should serve as the active site for the growth of BP crystals.

Besides, in order to find out if there were any other components at the active site, we studied the intersection point of BP by Raman spectroscopy. By comparing the obtained Raman spectra at the intersection point, the presence of Hittorf's phosphorus can be deduced (Figure S19). It should be pointed out that we also checked the products just near the intersection point by Raman spectroscopy. Only characteristic Raman peaks of BP crystals were observed (Figure S19). These results show that Hittorf's phosphorus may also play a certain role during the growth of BP crystals.

### Growth Mechanism of BP Crystals

**Figure 5:**
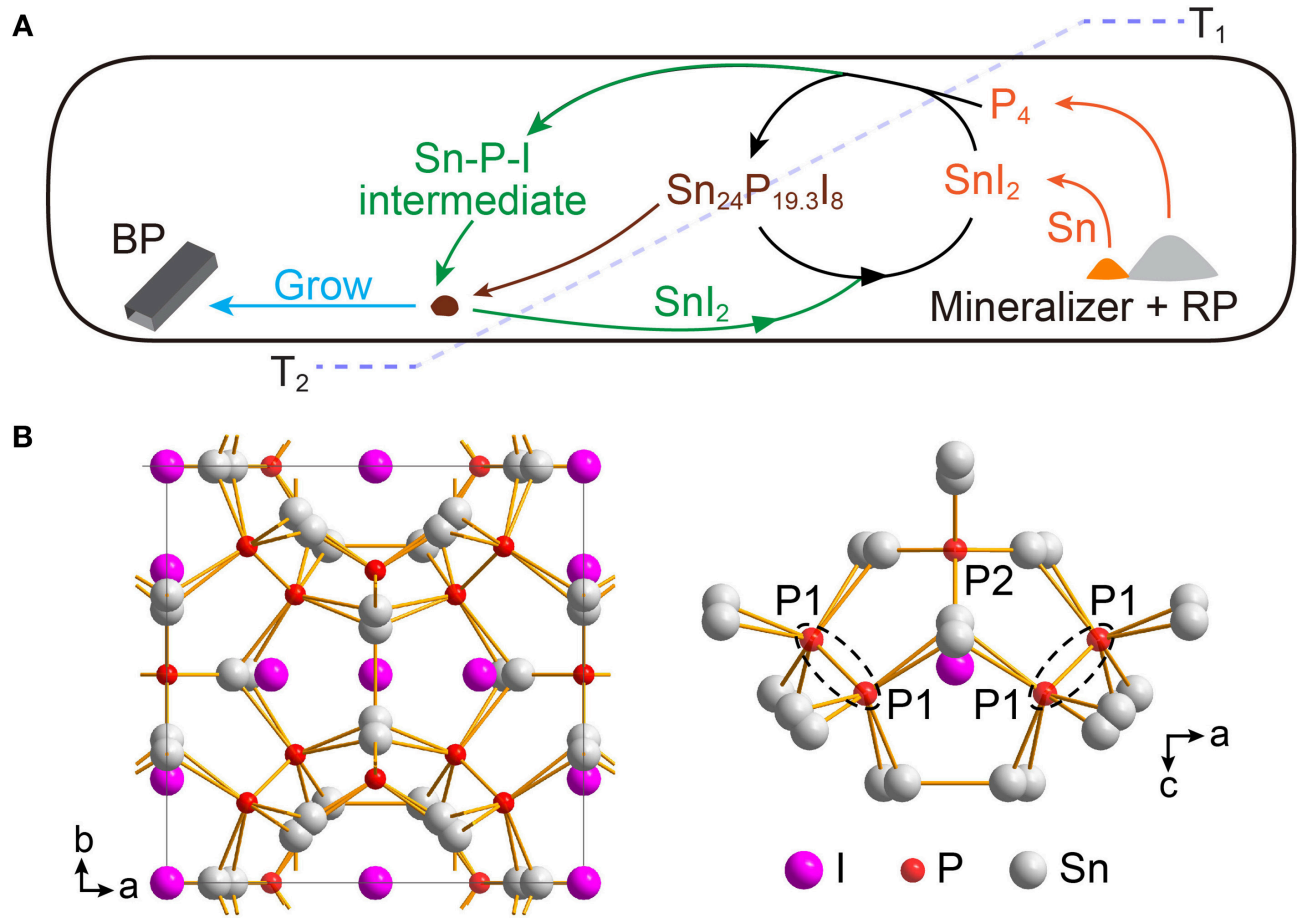
**(A)** Schematic illustration of the proposed mechanism of BP growth in the Sn-I assisted reaction. The mineralizer and red phosphorus (RP) first form SnI_2_, P_4_, and Sn at high temperature (T_1_) (orange colored arrows). The resulting SnI_2_, P_4_, and Sn then react to yield Sn_24_P_19.3_I_8_ which can deposit at the place with relatively low temperature (T_2_) (brown colored arrow). Subsequently, the BP crystals can grow with the assistance of SnI_2_ (green colored arrows). **(B)** Crystal structure of Sn_24_P_19.3_I_8_ where Sn and P atoms form a three dimensional framework with I atoms as guests.

As discussed in the former parts, in the Sn-I assisted synthesis of BP crystals, SnI_2_ should be a critical gaseous compound that may form gaseous intermediate to facilitate the growth of BP. Meanwhile, Sn_24_P_19.3_I_8_ should be the active site on which the BP crystals start to grow. Combining our results, the fundamentals of chemical vapor transport reactions (Binnewies et al., 2012), and previous proposed growth mechanisms of BP crystals together (Zhao et al., 2016a,b; Li et al., 2017; Zhang Z. et al., 2017; Shriber et al., 2018), we here propose the following formation mechanism of BP crystals in the Sn-I assisted reaction (Figure 5A).

At the beginning of the Sn-I assisted reaction, temperature increases for reactant sublimation. At this stage, despite different mineralizers, all reactants, placed at the right side of the ampoule in our studies (Figure 3), sublime or decompose gradually to generate gaseous SnI_2_, gaseous P_4_, and liquid Sn. The resulting gaseous SnI_2_, gaseous P_4_, and liquid Sn can further react to form Sn_24_P_19.3_I_8_ (Equation 1) and also probably some other Sn-P-I compounds at high temperature (e.g., ~650°C in this study).

(1) $$\begin{array}{r} 4{\text{SnI}}_{2}+20\text{Sn}+{4.825\text{P}}_{4}↔{\text{Sn}}_{24}{\text{P}}_{19.3}{\text{I}}_{8} \end{array}$$

Notably, in a sealed ampoule, the reaction (Equation 1), at high temperature, can be at equilibrium that is neither reactant-favored nor product-favored. Moreover, synthesis of Sn_24_P_19.3_I_8_ typically needs much longer time than that for preparing BP crystals (Shatruk et al., 1999). Therefore, we suggest that there are still some gaseous SnI_2_ in the reaction system. Regarding the liquid Sn presented at this stage, we believe that the slightly excess Sn can ensure the formation of Sn_24_P_19.3_I_8_ and gaseous SnI_2_.

After the temperature of the reaction system reaches 650°C, the reaction temperature starts to cool to 500°C with a low cooling rate. At this cooling stage, some Sn_24_P_19.3_I_8_ molecules deposit first at the place with a bit lower temperature because of vapor oversaturation. Remarkably, we here propose that Sn_24_P_19.3_I_8_, at the temperature of ~550–500°C, should be highly active as growth sites, due to its unique structure. Structurally, Sn_24_P_19.3_I_8_, with a clathrate type-I structure, is a three dimensional framework of tin and phosphorus atoms (Figure 5B, left panel), where I atoms are guests in the framework (Shatruk et al., 1999). There are two types of P atoms in the structure, and one type of P atoms appears in pairs with a P-P separation of 2.20 Å, similar to typical P-P bond (Figure 5B, right panel) (Shatruk et al., 1999). Furthermore, Sn_24_P_19.3_I_8_ crystals typically can have phosphorus vacancies in their structure (Li et al., 2017). Taking the TG analysis into account (Figure S13), we thus reason that the positions for P atoms in the Sn_24_P_19.3_I_8_ crystals, either P atoms or vacancies, can be the starting points for BP growth at the temperature of ~550–500°C.

After the formation of growth sites, BP crystals then can continuously grow with the assistance of SnI_2_, following a typical chemical vapor transportation process. More specifically, gaseous SnI_2_ molecules first react with gaseous P_4_ molecules at the high temperature zone, forming a Sn-P-I intermediate. The intermediate compounds then migrate to the zone with slightly lower temperature, where the growth sites are formerly formed. Subsequently, the intermediate compounds react with the active Sn_24_P_19.3_I_8_, yielding BP and releasing SnI_2_ for further reaction. The reaction would continue like polymerization until most of the P_4_ molecules are consumed. Finally, the gaseous SnI_2_ molecules are deposited randomly, mainly in the zone with lower temperature, when the temperature further decreases.

On a separate note, at the intersection points (growth sites) of the obtained BP crystals, we found Hittorf's phosphorus (Figure S19) which was also observed in some former studies (Chen et al., 2017; Zhang Z. et al., 2017). According to the experimental observations, Hittorf's phosphorus may function as the active sites or intermediate state for BP growth (Chen et al., 2017; Zhang Z. et al., 2017). However, considering that Hittorf's phosphorus can be directly obtained by heating red phosphorus in a vacuum container (Chen et al., 2017), we here believe that Hittorf's phosphorus should be the by-product formed at the beginning of BP growth.

## Conclusion

In conclusion, we have successfully synthesized BP crystals with various mineralizers, including SnI_4_/Sn, SnI_2_/Sn, Sn/I_2_, SnIP, and Sn_24_P_19.3_I_8_. By monitoring the Sn-I assisted reactions and characterizing the products, together with a series of control experiments, we here propose that BP crystals grow through a SnI_2_ mineralized reaction, and the Sn_24_P_19.3_I_8_ compound serves as an active site for BP growth. Although more detailed *in situ* studies are still needed, we believe that our studies shown here should provide insights into the growth mechanism of BP crystals, facilitating further manipulation of BP synthesis.

## Author Contributions

ML and XX conceived and designed the experiments. DW, PY, and LZ performed experiments. LW and HL contributed in the Raman analysis. ML, XX, LH, and WH supervised the research and contributed to the manuscript writing. All authors read and approved the final manuscript.

### Conflict of Interest Statement

The authors declare that the research was conducted in the absence of any commercial or financial relationships that could be construed as a potential conflict of interest.
