# Supplementary material for: Revisiting the Growth of Black Phosphorus in Sn-I Assisted Reactions
Source: Front Chem. 2019 Jan 28;7:21. doi: 10.3389/fchem.2019.00021 (PMC6362402; doi:10.3389/fchem.2019.00021)
Supplement: Supplementary file 1 [file Data_Sheet_1.pdf]

Supplementary material:

Revisiting the Growth of Black Phosphorus in Sn-I Assisted Reactions

Dongya Wang, Peng Yi, Lin Wang, Lu Zhang, Hai Li, Min Lu,* Xiaoji Xie,* Ling Huang, and Wei Huang

D. Wang, P. Yi, L. Wang, L. Zhang, Dr. M. Lu, Dr. X. Xie, Dr. L. Huang, Dr. W. Huang
Key Laboratory of Flexible Electronics (KLOFE), Institute of Advanced Materials (IAM), Nanjing Tech University (NanjingTech), Nanjing 211816, PR China
E-mail: iammlv@njtech.edu.cn, iamxjxie@njtech.edu.cn

Dr. W. Huang
Shaanxi Institute of Flexible Electronics, Northwestern Polytechnical University, Xi'an 710072, PR China

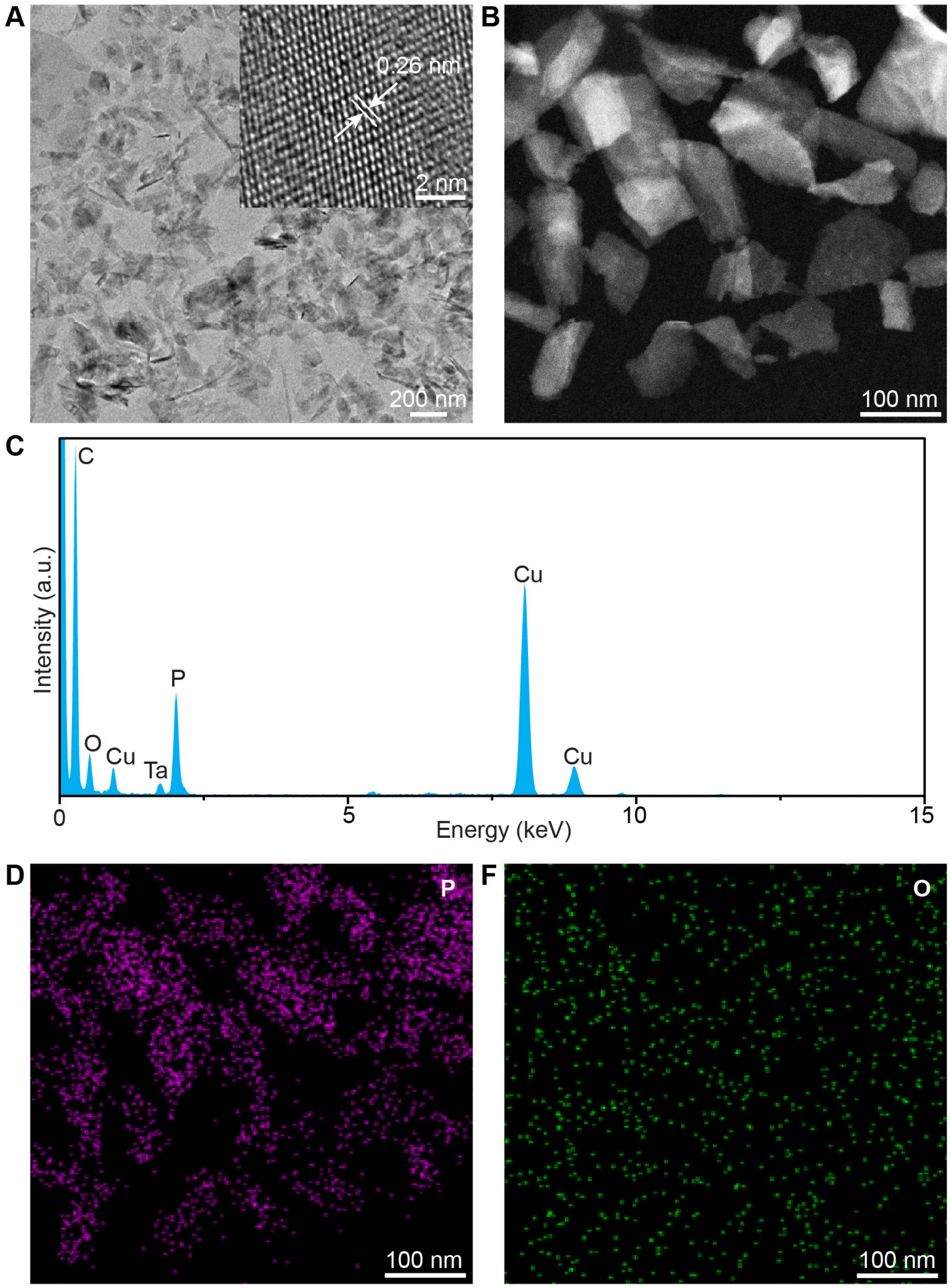

Figure S1. (**A**, **B**) TEM and dark-field STEM images of the exfoliated BP sheets, respectively. The inset in (**A**) is a high resolution TEM image of an exfoliated BP sheet. (**C**) Corresponding EDX spectrum and (**D**, **F**) elemental mapping of the exfoliated BP sheets shown in (**B**). The exfoliated BP sheets were obtained by the ultrasonic exfoliation method.[1] Notably, according to our control experiments, the appearance of other elements in (**C**), like C, Ta, O and Cu, should be due to the backgroup, including the TEM grid and sample holder. Meanwhile, the appearance of O element should be also partially due to the oxidation (degradation) of BP.

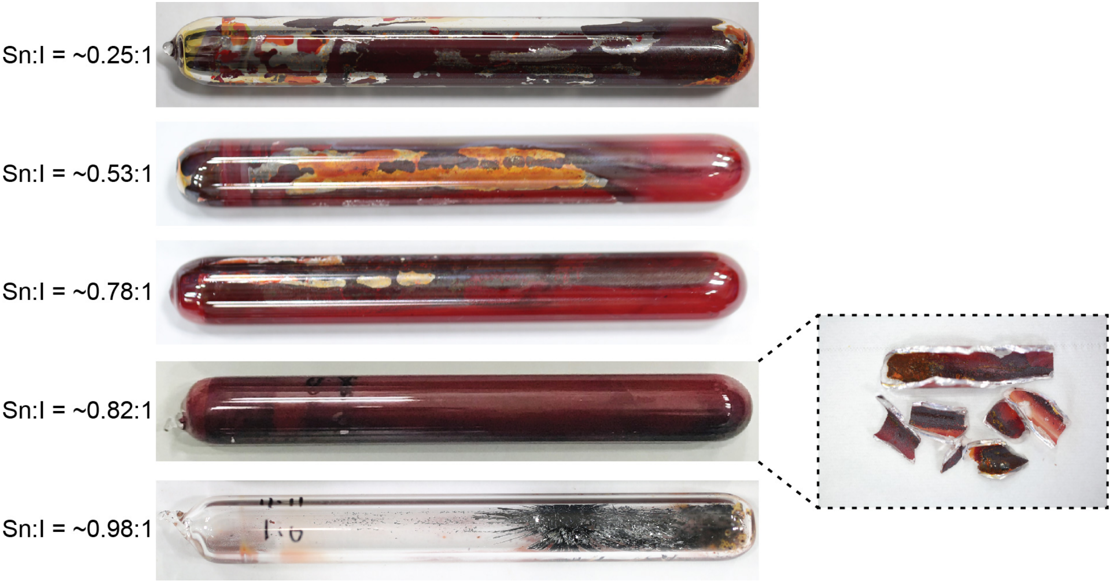

Figure S2. Representative photos of products obtained in the presence of the SnI_4/Sn mineralizers with different Sn:I molar ratios. If the Sn:I ratio was smaller than ~0.9:1, almost no BP crystal was found.

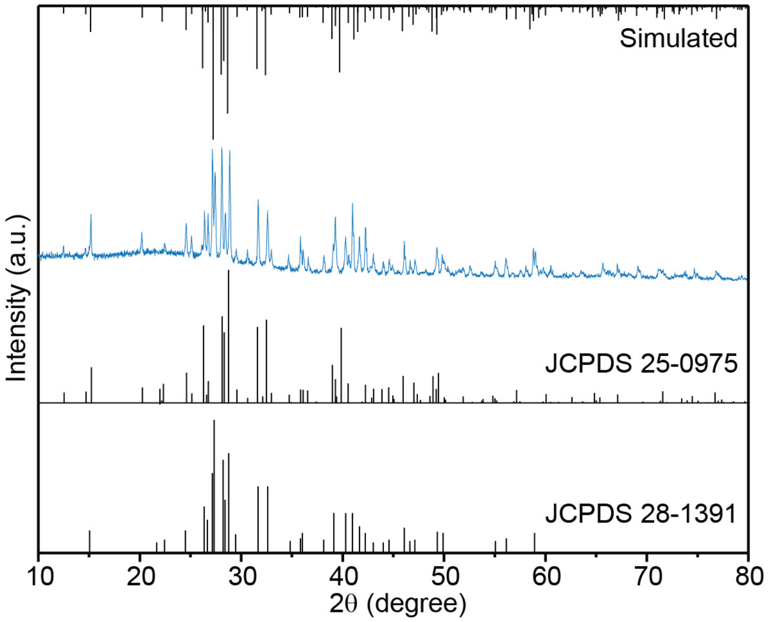

Figure S3. Powder XRD pattern of the as-synthesized SnI_2 (blue line). The diffraction pattern at the top is the simulated pattern according to the single crystal structure of SnI_2,[2] and the two diffraction patterns at the bottom are the literature references for SnI_2 (Joint Committee on Powder Diffraction Standards file number 25-0975 and 28-1391). The diffraction pattern of the as-synthesized SnI_2 is almost the same as the simulated one derived from the single crystal structure of SnI_2. Meanwhile, peak shifts, particularly at high angle, are observed, which can be attributed to the crystal strain. According to the literature references, we can conclude that the obtained SnI_2 powder is pure and may be a mixture of differently structured SnI_2.

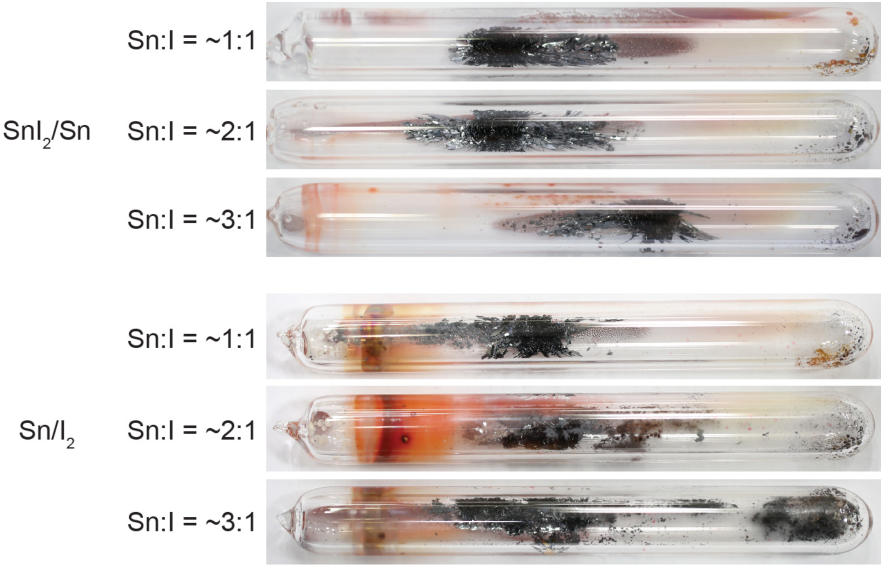

Figure S4. Representative photos of BP crystals obtained in the presence of SnI_2/Sn and Sn/I_2 mineralizers with different Sn:I molar ratios.

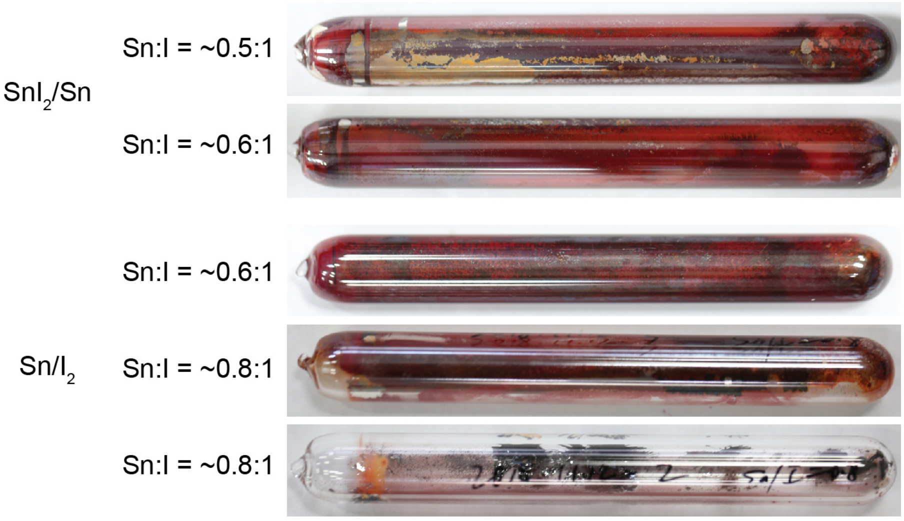

Figure S5. Representative photos of products obtained in the presence of SnI_2/Sn and Sn/I_2 mineralizers with different Sn:I molar ratios. According to our results, if the Sn:I ratio was set below ~0.9:1, in most of the cases, we did not get high quality BP crystals in high yield. However, occasionally, we got BP crystals when the Sn:I ratio was set near ~0.9:1 (the photo at the bottom of Figure S5). This may be due to the experimental deviations during weighting the mineralizer and losing of I containing compounds during sealing the ampoule.

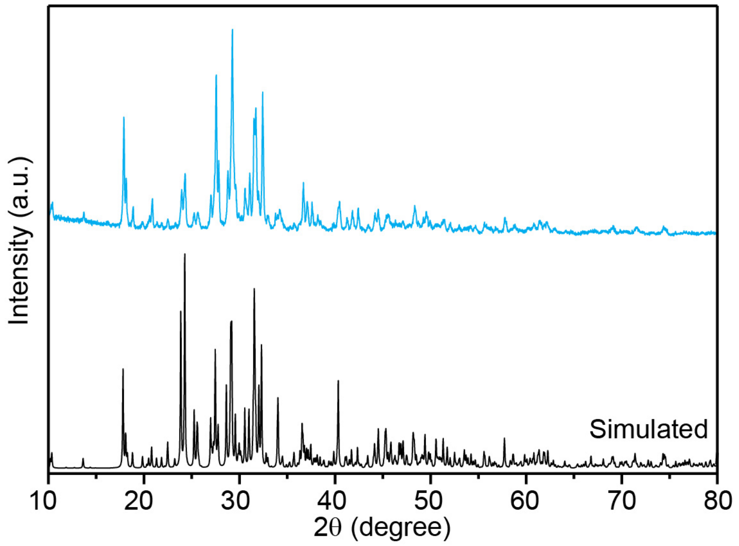

Figure S6. Powder XRD pattern of the as-synthesized SnIP (blue line). The diffraction pattern at the bottom is the simulated pattern according to the single crystal structure of SnIP.[3]

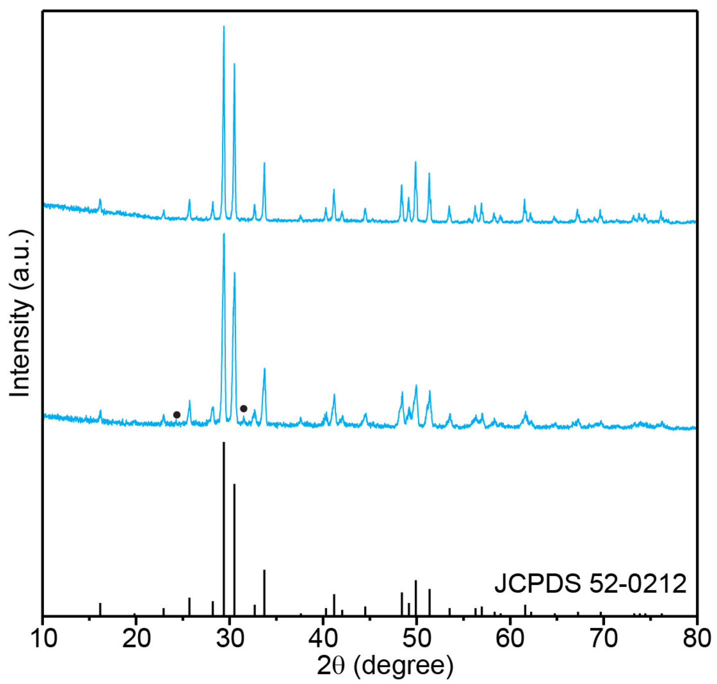

Figure S7. Powder XRD patterns of the as-synthesized $Sn_{24}P_{19.3}I_8$ (blue line). The diffraction pattern at the bottom is the literature reference for $Sn_{24}P_{19.3}I_8$ (Joint Committee on Powder Diffraction Standards file number 52-0212). Under our synthetic conditions, the XRD pattern of the resulting $Sn_{24}P_{19.3}I_8$ sometimes has small additional peaks at ~31.5 and 24 degree (marked by dark dots). The extra peaks can be attributed to the presence of SnIP (Figure S6). Notably, we compared the results by using pure $Sn_{24}P_{19.3}I_8$ and $Sn_{24}P_{19.3}I_8$ with impurities as mineralizers, and no difference was found.

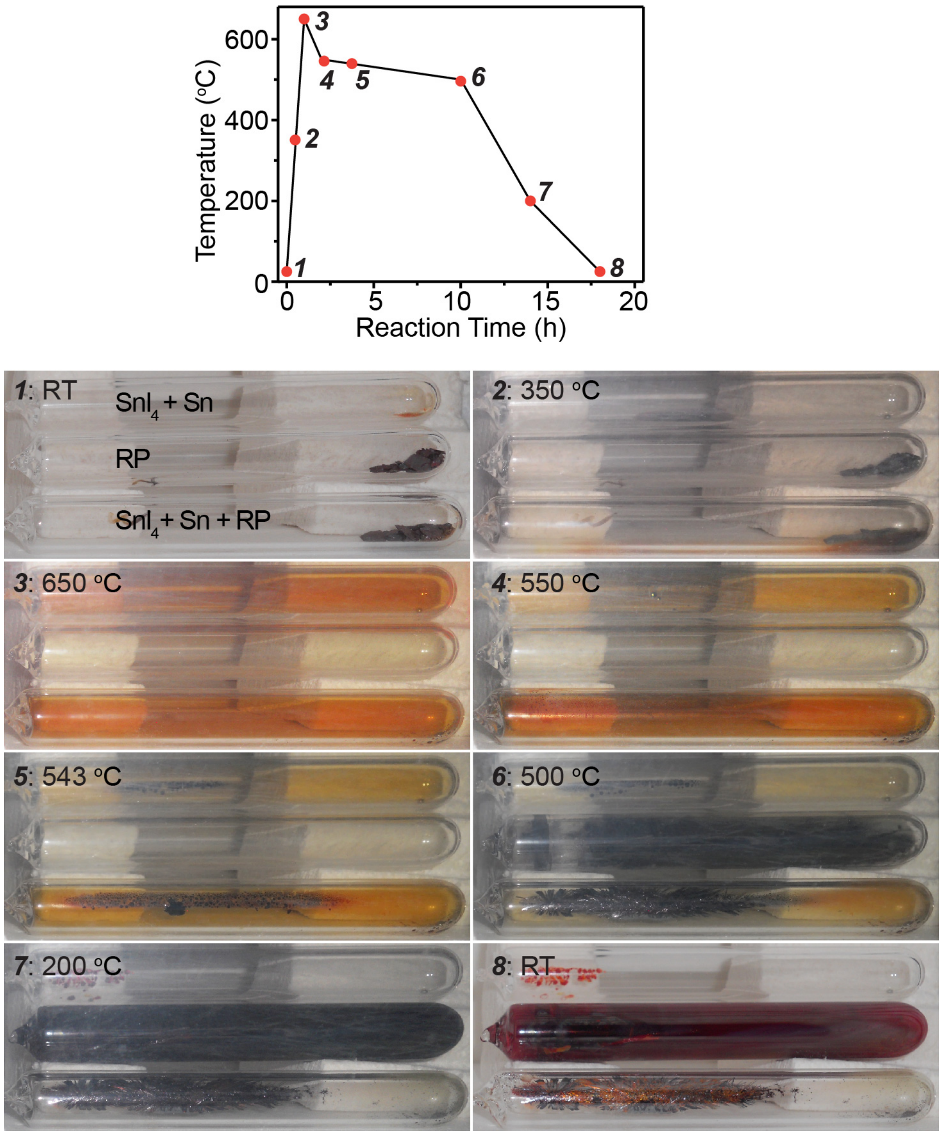

Figure S8. Typical time dependent temperature profile for synthesizing BP crystals (upper panel) and corresponding *in situ* photos taken during the synthesis. The red dots in the profile indicate the stages when the photos are taken. Note that RT and RP denote room temperature and red phosphorus, respectively.

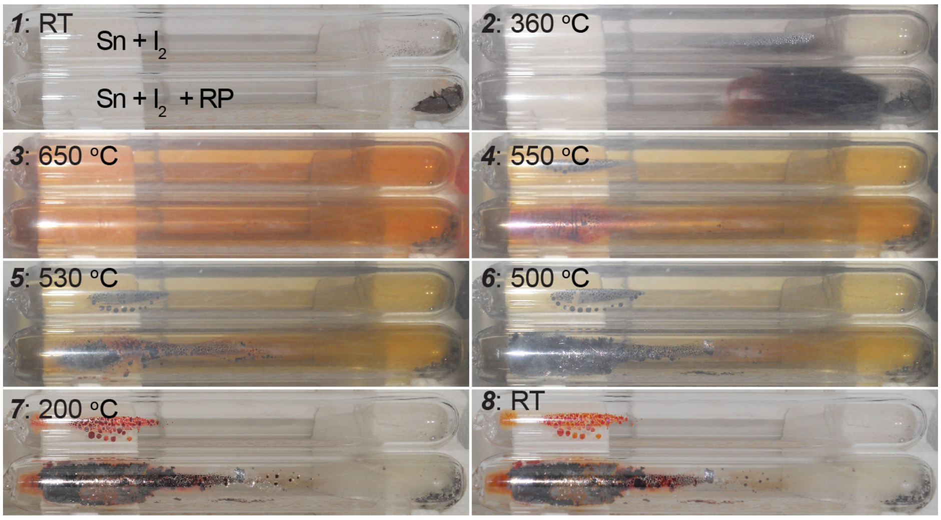

Figure S9. *In situ* photos taken during the synthesis of BP crystals in the presence of Sn/I_2 (Sn:I = ~3:1). Note that RT and RP denote room temperature and red phosphorus, respectively.

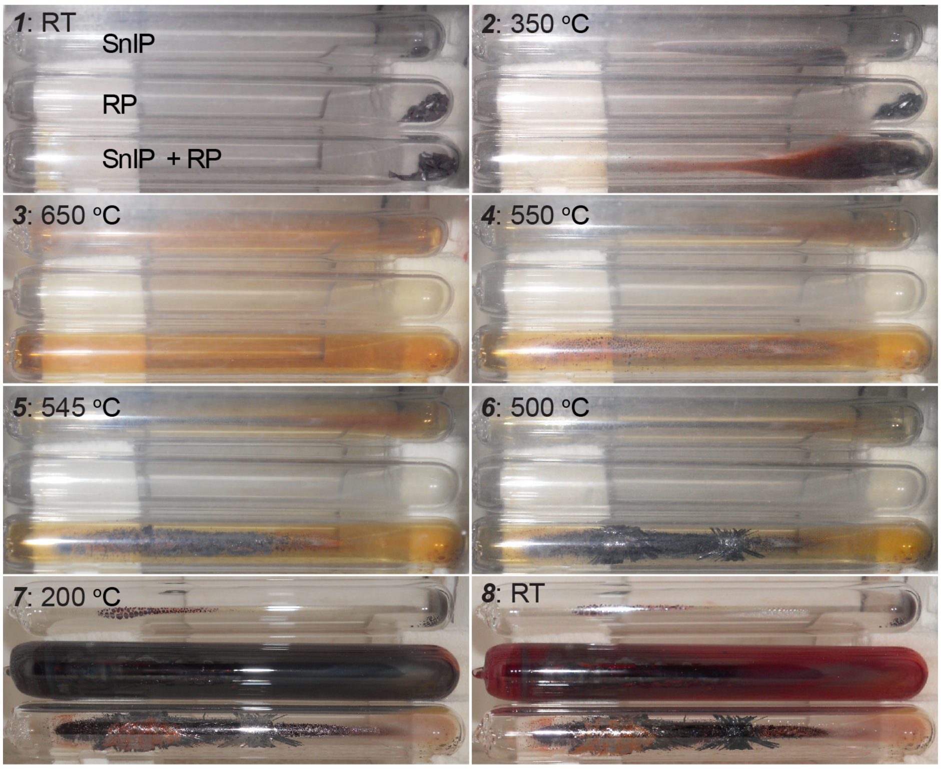

Figure S10. *In situ* photos taken during the synthesis of BP crystals in the presence of SnIP. Note that RT and RP denote room temperature and red phosphorus, respectively.

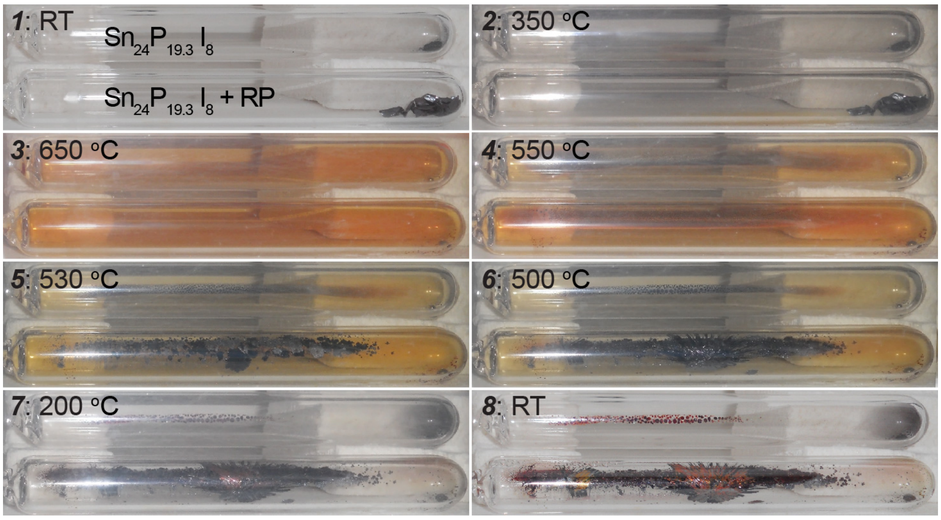

Figure S11. *In situ* photos taken during the synthesis of BP crystals in the presence of $Sn_{24}P_{19.3}I_8$. Note that RT and RP denote room temperature and red phosphorus, respectively.

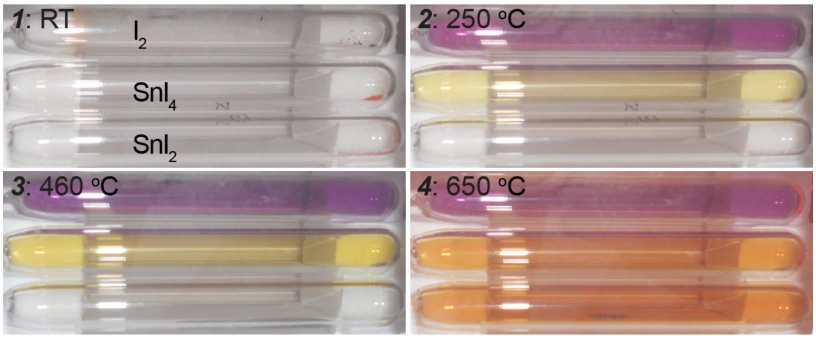

Figure S12. Photos of ampoules with I_2 (5 mg), SnI_4 (6.3 mg), and SnI_2 (7.7 mg) under different temperatures. Note that RT denotes room temperature.

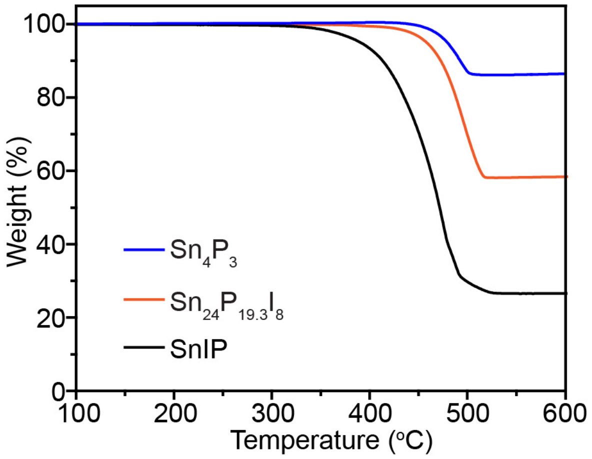

Figure S13. Thermogravimetric profiles of Sn_4P_3, $Sn_{24}P_{19.3}I_8$, and SnIP, respectively.

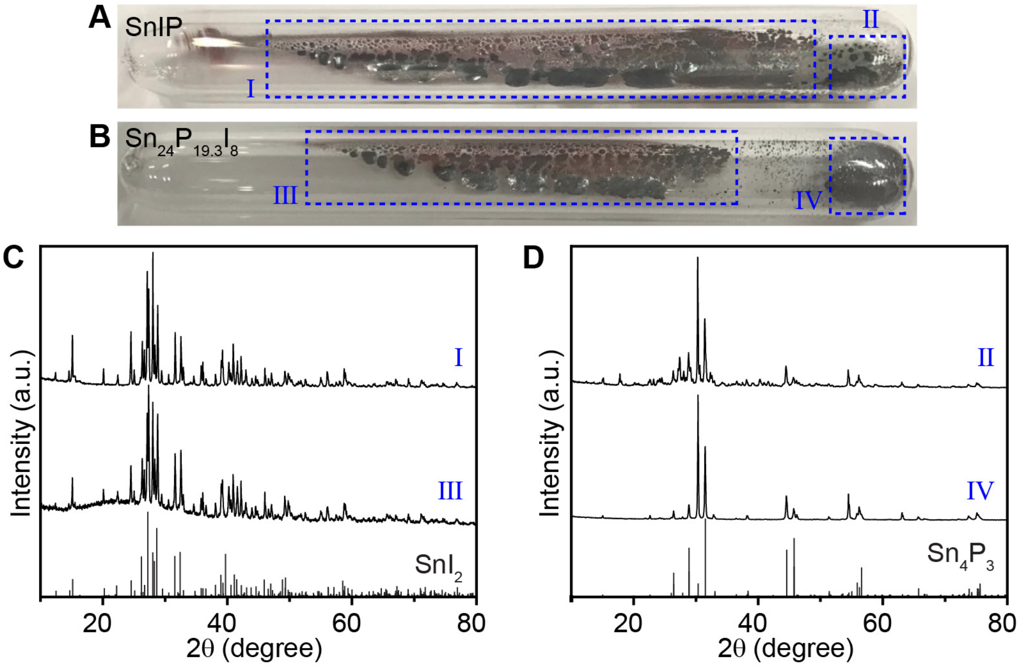

Figure S14. **(A, B)** Photos of SnIP and $Sn_{24}P_{19.3}I_8$-containing ampoules that experienced a programmed heating as that for synthesizing BP crystals, respectively. **(C, D)** Powder XRD patterns of solids collected from the locations labelled as I, II, III and IV in **(A)** and **(B)**. The diffraction patterns at the bottom of **(C)** and **(D)** are the literature references for SnI_2 (the simulated pattern according to the single crystal structure of SnI_2[2]) and Sn_4P_3 (the simulated pattern according to the single crystal structure of Sn_4P_3[4]), respectively.

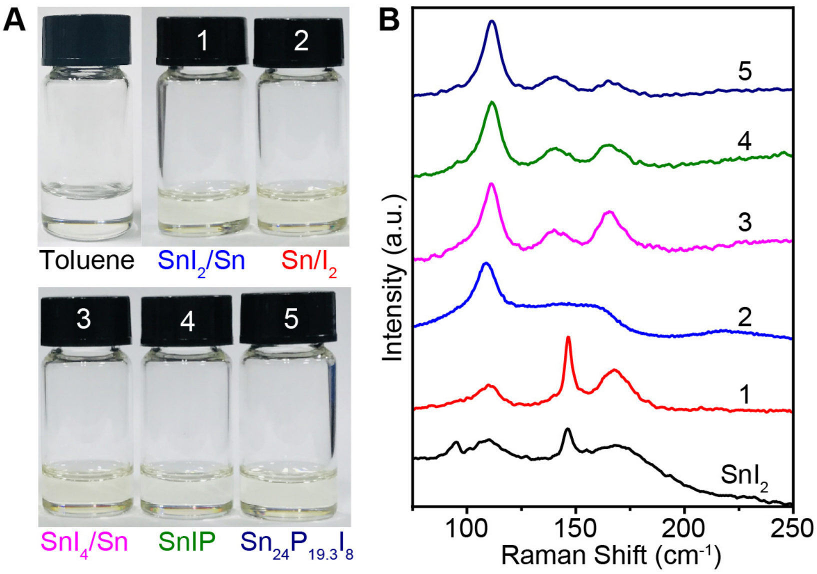

Figure S15. (A) Photos of pure toluene and toluene solutions after washing the as-synthesized BP crystals prepared in the presence of different mineralizers. **(B)** Raman spectra of as-synthesized SnI_2 and the solid obtained by distilling the toluene solutions shown in **(A)**.

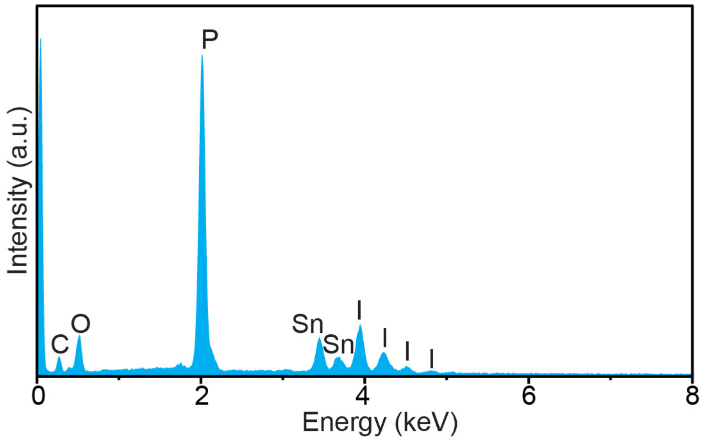

Figure S16. Representative EDX spectrum of the intersection point in the as-prepared BP crystals before thoroughly washed by hot toluene. Accordingly, the molar ratio of Sn:I was determined as ~0.6:1. Note that the EDX spectrum was obtained in the scanning electron microscope equipped with an energy dispersive spectrometer.

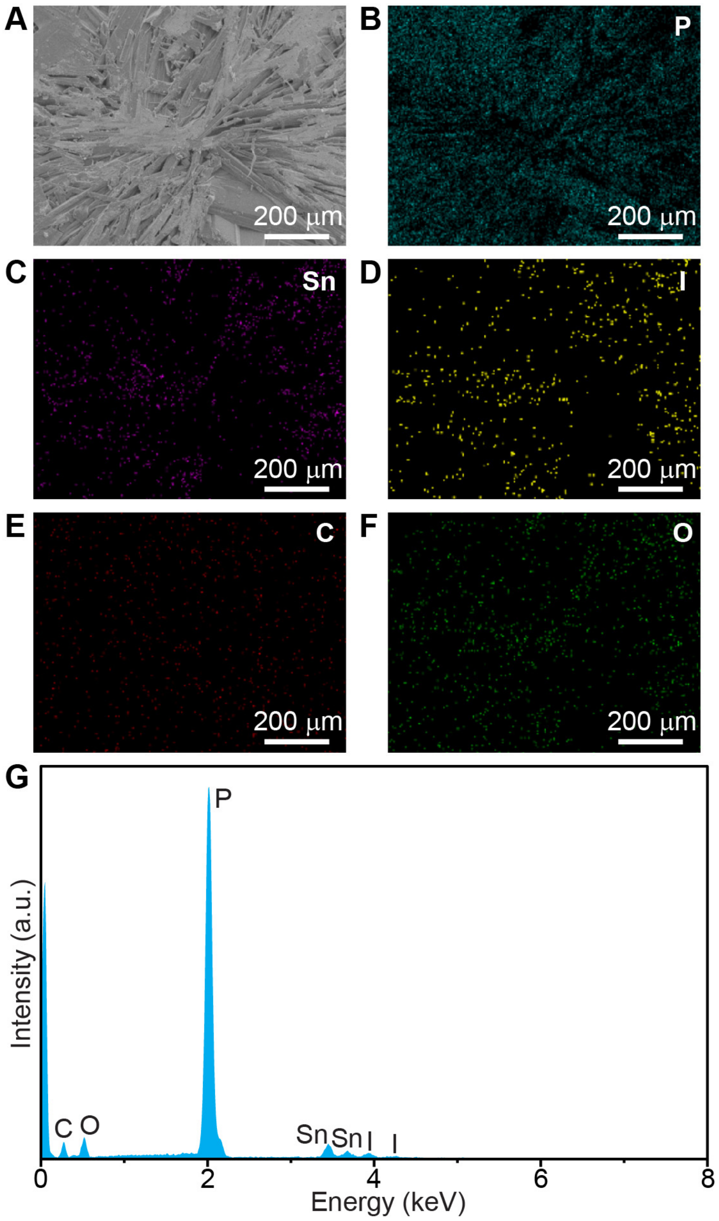

Figure S17. (**A**) SEM image of a representative intersection point in the as-prepared BP crystals after thoroughly washed by hot toluene. (**B-F**) Corresponding elemental mapping of the intersection point shown in (**A**). (**G**) Corresponding EDX spectrum of the intersection point shown in (**A**).

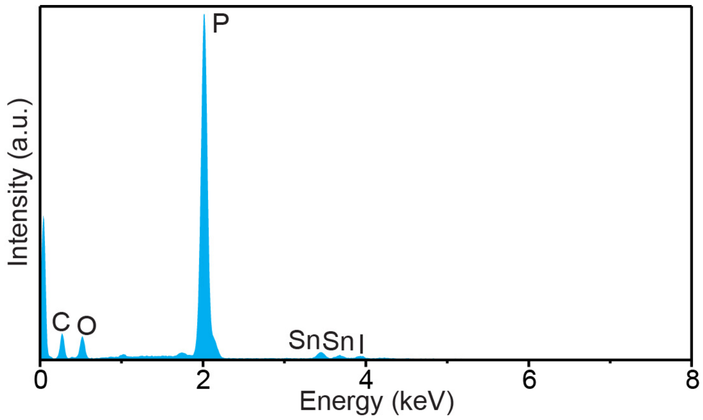

Figure S18. Representative EDX spectrum of the intersection point in the BP crystals synthesized in the presence of $Sn_{24}P_{19.3}I_8$. Accordingly, the molar ratio of Sn:I was determined as ~2.9:1. Note that the BP crystals were washed thoroughly by hot toluene, and the EDX spectrum was obtained in the scanning electron microscope equipped with an energy dispersive spectrometer.

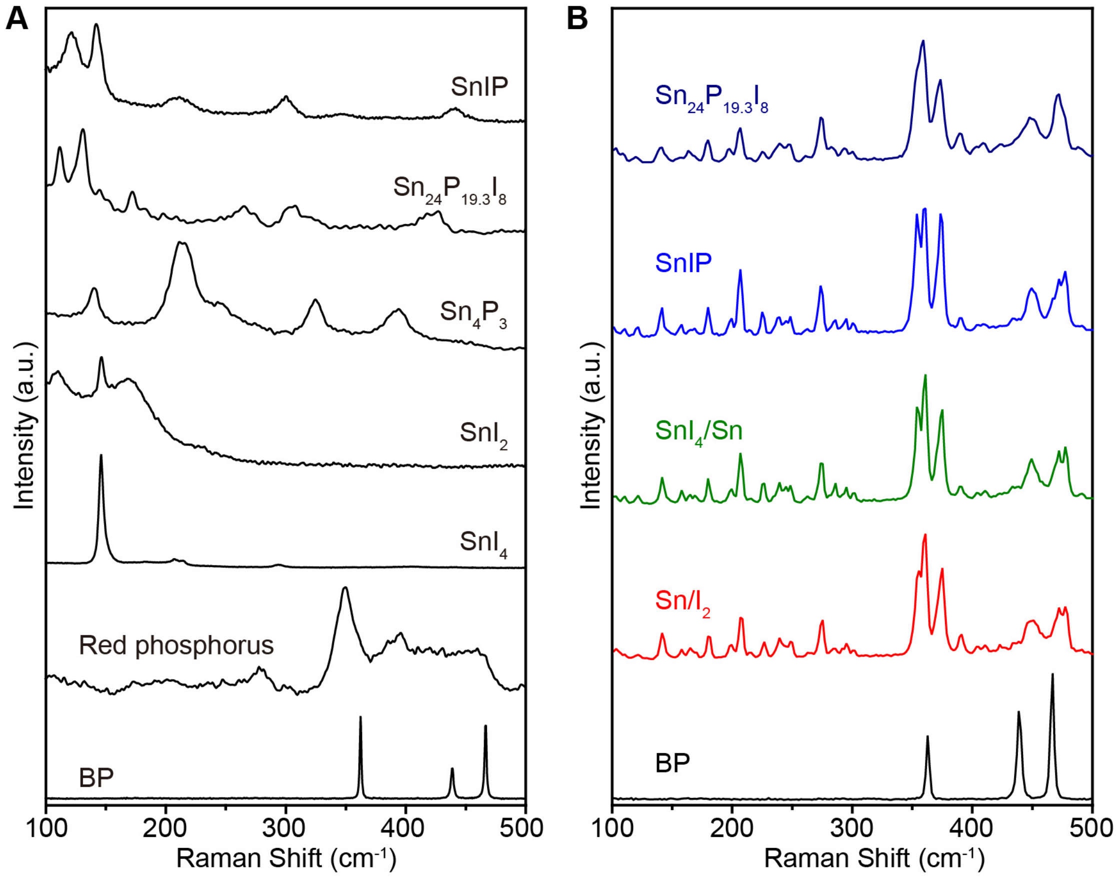

Figure S19. (A) Raman spectra of black phosphorus (BP), red phosphorus, as-synthesized SnI_4, SnI_2, Sn_4P_3, $Sn_{24}P_{19.3}I_8$ and SnIP. **(B)** Raman spectra of the intersection points of BP crystals synthesized in the presence of different mineralizers (Sn/I_2, SnI_4/Sn, SnIP, and $Sn_{24}P_{19.3}I_8$). The Raman spectrum at the bottom of **(B)** is the Raman spectrum of a randomly selected place near the intersection point (BP crystals prepared in the presence of $Sn_{24}P_{19.3}I_8$). This spectrum indicates that the products, just near the intersection point, are pure BP. It should be mentioned that the as-synthesized BP crystals were washed thoroughly with hot toluene before analysis.

The Raman spectra of the intersection points cannot match the Raman spectra of black phosphorus, red phosphorus, Sn-I compounds or Sn-P-I compounds (Figure S19A). After carefully comparing the Raman spectra with literatures,[5,6] we can conclude that there is some Hittorf's phosphorus at the intersection points, although the presence of other compounds cannot be ruled out. Typically, Hittorf's phosphorus can be directly obtained by heating red phosphorus in a vacuum container without any additives.[7] In contrast, black phosphorus currently can only be obtained in the presence of Sn-I containing additives under similar conditions. In addition, some recent studies reveal that Hittorf's phosphorus can form in the reaction system, while black phosphorus crystals can grow at the sites away from Hittorf's phosphorus.[7,8] Together with our experimental results, we therefore deduce that Hittorf's phosphorus should not be the nucleation site for the growth of black phosphorus.

Reference

[1] Yang, X., Wang, D., Shi, Y., Zou, J., Zhao, Q., Zhang, Q., Huang, W., Shao, J., Xie, X., and Dong, X. (2018). Black phosphorus nanosheets immobilizing Ce6 for imaging-guided photothermal/photodynamic cancer therapy. *ACS Appl. Mater. Interfaces* 10, 12431-12440. doi: 10.1021/acsami.8b00276

[2] Howie, R. A., Moser, W., and Trevena, I. C. (1972). The crystal structure of tin(II) iodide. *Acta Crystallogr. Sect. B* 28, 2965-2971. doi: 10.1107/S0567740872007290

[3] Pfister, D., Schäfer, K., Ott, C., Gerke, B., Pöttgen, R., Janka, O., Baumgartner, M., Efimova, A., Hohmann, A., Schmidt, P., Venkatachalam, S., van Wüllen, L., Schürmann, U., Kienle, L., Duppel, V., Parzinger, E., Miller, B., Becker, J., Holleitner, A., Weihrich, R., and Nilges, T. (2016). Inorganic double helices in semiconducting SnIP. *Adv. Mater.* 28, 9783-9791. doi: 10.1002/adma.201603135

[4] Kuz'Ma, Yu. B., Chikhrii, S. I., and Davydov, V. N. (1999). Refined crystal structure of Sn_4P_3. *Neorg. Mater.* 35, 17-18.

[5] Baumer, F., Ma, Y., Shen, C., Zhang, A., Chen, L., Liu, Y., Pfister, D., Nilges, T., and Zhou, C. (2017). Synthesis, characterization, and device application of antimony-substituted violet phosphorus: a layered material. *ACS Nano* 11, 4105-4113. doi: 10.1021/acsnano.7b00798

[6] Zhang, Z., Xing, D.-H., Li, J., and Yan, Q. (2017). Hittorf's phosphorus: the missing link during transformation of red phosphorus to black phosphorus. *CrystEngComm* 19, 905-909. doi: 10.1039/C6CE02550A

[7] Chen, Z., Zhu, Y., Lei, J., Liu, W., Xu, Y., and Feng, P. (2017). A stage-by-stage phase-induction and nucleation of black phosphorus from red phosphorus under low-pressure mineralization. *CrystEngComm* 19, 7207-7212. doi: 10.1039/c7ce01492a

[8] Zhao, M., Qian, H., Niu, X., Wang, W., Guan, L., Sha, J., and Wang, Y. (2016). Growth mechanism and enhanced yield of black phosphorus microribbons. *Cryst. Growth Des.* 16, 1096-1103. doi: 10.1021/acs.cgd.5b01709
